# Marine Alga *Ecklonia cava* Extract and Dieckol Attenuate Prostaglandin E_2_ Production in HaCaT Keratinocytes Exposed to Airborne Particulate Matter

**DOI:** 10.3390/antiox8060190

**Published:** 2019-06-21

**Authors:** Jae Won Ha, Hyerim Song, Seong Su Hong, Yong Chool Boo

**Affiliations:** 1 Department of Molecular Medicine, School of Medicine, Kyungpook National University, Daegu 41944, Korea; jaewon1226@knu.ac.kr (J.W.H.); happyhyerim@knu.ac.kr (H.S.); 2 BK21 Plus KNU Biomedical Convergence Program, Kyungpook National University, Daegu 41944, Korea; 3 Cell and Matrix Research Institute, Kyungpook National University, Daegu 41944, Korea; 4 Bio-Center, Gyeonggido Business & Science Accelerator (GBSA), Suwon 16229, Korea; bestgene@gbsa.or.kr

**Keywords:** *Ecklonia cava* Kjellman, dieckol, airborne particulate matter, keratinocytes, prostaglandin E_2_, cyclooxygenase, prostaglandin E_2_ synthase

## Abstract

Atmospheric particulate matter (PM) is an important cause of skin damage, and an increasing number of studies have been conducted to discover safe, natural materials that can alleviate the oxidative stress and inflammation caused by PM. It has been previously shown that the extract of *Ecklonia cava* Kjellman, a perennial brown macroalga, can alleviate oxidative stress in epidermal keratinocytes exposed to PM less than 10 microns in diameter (PM10). The present study was undertaken to further examine the anti-inflammatory effects of *E. cava* extract and its major polyphenolic constituent, dieckol. HaCaT keratinocytes were exposed to PM10 in the presence or absence of *E. cava* extract or dieckol and analyzed for their viability, prostaglandin E_2_ (PGE_2_) release, and gene expression of cyclooxygenase (COX)-1, COX-2, microsomal prostaglandin E_2_ synthase (mPGES)-1, mPGES-2, and cytosolic prostaglandin E_2_ synthase (cPGES). PM10 treatment decreased cell viability and increased the production of PGE_2_, and these changes were partially abrogated by *E. cava* extract. *E. cava* extract also attenuated the expression of COX-1, COX-2, and mPGES-2 stimulated by PM10. Dieckol attenuated PGE_2_ production and the gene expression of COX-1, COX-2, and mPGES-1 stimulated by PM10. This study demonstrates that *E. cava* extract and dieckol alleviate airborne PM10-induced PGE_2_ production in keratinocytes through the inhibition of gene expression of COX-1, COX-2, mPGES-1, and/or mPGES-2. Thus, *E. cava* extract and dieckol are potentially useful natural cosmetic ingredients for counteracting the pro-inflammatory effects of airborne PM.

## 1. Introduction

The World Health Organization (WHO) reported that more than 4.2 million people died in 2018 from air pollution, making it the largest single environmental health risk factor (https://www.who.int). Air pollutants causing serious health risks include particulate matter (PM), ozone (O_3_), nitrogen dioxide (NO_2_), and sulphur dioxide (SO_2_) [1]. PM, the main component of air pollution, is composed of inorganic and organic, solid, and liquid particles suspended in the air [2]. PM can be produced directly or indirectly from several sources including agriculture, industry, power plants, automobiles, construction, and forest fires [3]. PM less than 10 and 2.5 microns in diameter (PM10 and PM2.5) can penetrate deep into the lungs and enter the bloodstream. Exposure to PM increases the incidence of cardiovascular, cerebrovascular, and respiratory diseases [4,5,6,7].

The skin is a barrier between the body and the outer environment and is directly exposed to harmful environmental pollutants. Patients with compromised skin barriers are more affected by PM through increased absorption thereof by the percutaneous tract [8,9]. PM itself can impede the barrier function, enhancing subsequent drug absorption [10]. PM that infiltrates the skin can aggravate skin diseases, such as atopic dermatitis, acne, and psoriasis [11]. PM is also associated with premature skin aging [12] and hyperpigmentation [13]. Simultaneous PM and UV ray exposure synergistically exert negative effects on the skin and can lead to photo-aging and cancer [14,15]. 

Airborne PM has been implicated in the production of reactive oxygen species (ROS) and the expressions of cytokines and matrix metalloproteinases involved in oxidative stress and inflammation, as demonstrated in human dermal fibroblasts, epidermal keratinocytes, and reconstructed epidermis models [16,17,18,19]. PM increases the production of the eicosanoid mediator prostaglandin (PG) E_2_ and decreases filaggrin expression in human keratinocytes, leading to reduced skin barrier function [20,21]. In contrast, eupafolin, derived from the medicinal herb *Phyla nodiflora*, inhibited PM-induced cyclooxygenase (COX)-2 expression and PGE_2_ production in HaCaT keratinocytes [22]. Resveratrol, a polyphenol found in grapes and red wine, reduced PM-induced COX-2 expression and PGE_2_ production in human fibroblast-like synoviocytes [23]. Therefore, dermatological and cosmetic approaches using safe and effective antioxidants might alleviate the adverse skin reactions that arise from PM exposure [24].

*Ecklonia cava* Kjellman, which belongs to the Laminariaceae family, is a perennial brown macroalga widely distributed along the coast of Korea and is used in traditional medicine [25]. *E. cava* contains phlorotannins such as eckol and dieckol [26] and has been reported to have antioxidant, anti-inflammatory, antibacterial, antidiabetic, and anticancer properties [27,28,29,30,31,32]. In a previous study, this laboratory showed that *E. cava* extract and dieckol attenuated lipid peroxidation and the expression of inflammatory cytokines in human keratinocytes exposed to PM10 [33]. Building on this previous work, we further examined here whether *E. cava* extract and dieckol affect PM10-induced PGE_2_ release and the gene expression of enzymes involved in the synthesis of PGE_2_ in human keratinocytes. 

## 2. Materials and Methods 

### 2.1. Marine Alga Extracts

The extracts of 50 different marine algae were purchased from Jeju Biodiversity Research Institute of Jeju Technopark (Jeju, Korea), as previously reported [34]. 

### 2.2. Purification of Dieckol from E. cava

Dried *E. cava* was purchased from Jayeoncho (http://www.jherb.com) (Seoul, Korea) and 200 g of powder was extracted with 1.0 L 80% *v/v* aqueous ethanol for 7 days at room temperature (usually 25 °C). The slurry was then filtered through a Whatman No. 1 filter paper (Sigma–Aldrich, St. Louis, MO, USA) and the filtrate was evaporated under reduced pressure, yielding 18 g crude extract. The crude extract was dispersed in 0.2 L water and partitioned with organic solvents, yielding 1.49 g methylene chloride fraction, 2.83 g ethyl acetate fraction, 3.46 g 1-butanol fraction, and 8.65 g water fraction. A portion of the ethyl acetate fraction (2.45 g) was further fractionated by normal phase chromatography on a ϕ3 cm × 20 cm column of silica gel (Sigma–Aldrich) and eluted with a 4:1 *v/v* mixed solvent of methylene chloride and methanol (MeOH). Fractions that contained a significant amount of dieckol were combined and evaporated under reduced pressure, yielding 0.81 g of dry materal. This material was subjected to reversed phase chromatography on a ϕ3 cm × 20 cm column of YMC-GEL ODS-A (YMC Co., Ltd., Kyoto, Japan) and eluted using stepped-gradient 30–70% *v/v* aqueous MeOH. The fractions that contained dieckol were pooled and evaporated under reduced pressure to dryness, yielding 60 mg of compound **1** (purity, 97%). 

### 2.3. Instrumental Analysis

Nuclear magnetic resonance (NMR) spectra were obtained using a Bruker Ascend III 700 (CryoProbe) spectrometer (Bruker BioSpin, Rheinstetten, Germany). Chemical shifts in δ values were referenced to an internal standard, tetramethylsilane (TMS). Electrospray ionization mass spectra (ESI-MS) were obtained using a TSQ Quantum Discovery MAX (Thermo Fisher Scientific Inc., Waltham, MA, USA).

Compound **1** (dieckol): pale brown powder; UV (MeOH) λmax (log ε_M_), 204 nm (5.19), 232 nm (5.05), 291 nm (3.97); ^1^H-NMR (CD_3_OD, 700 MHz) and ^13^C-NMR (CD_3_OD, 175 MHz) data, Table 1; ESI-MS in negative ion mode, *m*/*z* 741.61 [M − H]^−^, calculated *m/z* 742.55 [M]^−^ (C_36_H_22_O_18_).

### 2.4. High Performance Liquid Chromatography (HPLC)

HPLC was carried out using a Waters Alliance HPLC System (Waters, Milford, MA, USA.) consisting of a Waters e2695 Separation Module and a Waters 2996 photodiode array detector. The stationary phase was a 5 μm, 4.6 mm × 250 mm Hector-M C_18_ column (RS Tech Co., Daejeon, Korea), and the mobile phase was a gradienet mixture of 0.1% phosphoric acid (A) and acetonitrile (B). The solvent gradient program was as follows: 0–30 min, a linear gradient from 0–100% B; 30–40 min, 100% B. The flow rate of the mobile phase was 0.6 mL min^−1^. 

### 2.5. Cell Culture

HaCaT cells, an immortalized human keratinocyte cell line established by Norbert E. Fusenig [35], and so named so to denote its origin from human adult skin keratinocytes propagated under low Ca^2+^ conditions and elevated temperature, were obtained from In-San Kim (Kyungpook National University, Daegu, Korea) [36]. Cells were cultured in a closed incubator at 37 °C in humidified air containing 5% CO_2_. Cells were administered a DMEM/F-12 medium (GIBCO-BRL, Grand Island, NY, USA) containing 10% fetal bovine serum, 100 U mL^−1^ penicillin, 100 μg mL^−1^ streptomycin, 0.25 μg mL^−1^ amphotericin B, and 10 μg mL^−1^ hydrocortisone every three days. 

### 2.6. Treatment of Cells with PM10 

The cells were plated onto 6-well culture plates (SPL Life Sciences, Pocheon, Korea) at 8 × 10^4^ cells/well and cultured in a growth medium for 24 h. A standardized PM_10_-like fine dust (European Reference Material ERM-CZ120PM10) (Sigma–Aldrich) was suspended in phosphate-buffered saline (PBS) at 100 times the final concentration of each treatment before each experiment. Cells were treated with PM10 at specific concentrations ranging from 25 to 400 μg mL^−1^ for 24 to 48 h, depending on the experimental purpose, with or without *E. cava* extract or dieckol at specified concentrations. N-acetyl cysteine (NAC) (Sigma–Aldrich) was used as a positive control antioxidant.

### 2.7. Cell Viability Assay

Cell viability was assessed using a 3-[4,5-dimethylthiazol-2-yl]-2,5-diphenyl tetrazolium bromide (MTT) assay. Cells were incubated in 200 μL growth medium containing 1 mg mL^−1^ MTT (Amresco, Solon, OH, USA) for 2 h at room temperature. After removing the medium, cells were extracted with 200 μL dimethyl sulfoxide, and absorbances of the formazan dye were determined at 595 nm with a SPECTROstar Nano microplate reader (BMG LABTECH GmbH, Ortenberg, Germany).

### 2.8. Enzyme-Linked Immunosorbent Assay (ELISA)

Levels of PGE_2_ protein in the culture medium were determined using a prostaglandin E_2_ express ELISA kit (Cayman Chemical Co., Ann Arbor, MI, USA). In this assay, a fixed amount of PGE_2_-acetylcholinesterase (AChE) conjugate is used as a PGE_2_ tracer whose binding to PGE_2_ monoclonal antibody is inversely proportional to the amount of PGE_2_ derived from the sample. Briefly, 50 µL of 4-fold-diluted cell culture media or standard PGE_2_ solutions were transferred to microplate wells containing immobilized goat polyclonal anti-mouse IgG. PGE_2_ tracer and PGE_2_ monoclonal antibody were then added to each well, and the mixtures were incubated at 4 °C for 18 h. The well was rinsed 5 times with wash buffer and Ellman’s reagent containing acetylthiocholine and 5,5′-dithio-bis-(2-nitrobenzoic acid) was added to initiate the AChE reaction. After 60 min, absorbances were measured at 405 nm with a SPECTROstar Nano microplate reader (BMG LABTECH GmbH). The amount of PGE_2_ was estimated using a standard curve.

### 2.9. Quantitative Reverse-Transcriptase Polymerase Chain Reaction (qRT-PCR) Analysis

The mRNA levels of COX-1, COX-2, microsomal prostaglandin E_2_ synthase (mPGES)-1, mPGES-2, and cytosolic prostaglandin E_2_ synthase (cPGES) were determined by qRT-PCR using a StepOnePlus™ Real-Time PCR System (Applied Biosystems, Foster City, CA, USA). Total RNA was extracted from cells with an RNeasy kit (Qiagen, Valencia, CA, USA), and this RNA was used as a template for the synthesis of complementary DNA (cDNA) with a high capacity cDNA archive kit (Applied Biosystems). Gene-specific primers for qRT-PCR analysis were purchased from Macrogen (Seoul, Korea), and their nucletotide sequences are shown in Table 2. The qRT-PCR reaction mixture (20 μL) consisted of SYBR^®^ Green PCR Master Mix (Applied Biosystems), cDNA (60 ng), and gene-specific primer sets (2 pmole). Thermal cycling parameters were set as follows: 50 °C for 2 min, 95 °C for 10 min, 40 amplification cycles of 95 °C for 15 s and 60 °C for 1 min, and a dissociation step. In each run, the melting curve analysis confirmed homogeneity of the PCR product. The mRNA levels of each gene were calculated relative to that of the internal reference, glyceraldehyde 3-phosphate dehydrogenase (GAPDH), using the comparative Ct method [37]. Ct is defined as the number of cycles required for the PCR signal to exceed the threshold level. Fold changes in the test group compared to the control group were calculaed as 2^−ΔΔCt^, where ΔΔCt = ΔCt_(test)_ − ΔCt_(control)_ = [Ct_(gene, test)_ − Ct_(reference, test)_] − [Ct_(gene, control)_ − Ct_(reference, control)_].

### 2.10. Assay for Cellular ROS Production

Cellular ROS production was assessed by using 2′-7′-dichlorodihydrofluorescein diacetate (DCFH-DA), a cell permeable fluorescent probe sensitive to changes in the redox state of a cell [41]. The cells were plated onto 12-well culture plates (SPL Life Sciences) at 4 × 10^4^ cells/well for 24 h. Cells were pre-labeled with 10 μM DCFH-DA (Sigma-Aldrich) for 30 min and treated with 100 μg mL^−1^ PM10 alone or in combination with a test material at different concentrations for 30 min. Cells were extracted with 20 mM Tris-Cl buffer (pH 7.5) containing 1% sodium dodecyl sulfate (SDS) and 2.5 mM ethylenediamine-N,N,N′,N′-tetraacetic acid (EDTA) (150 μL/well). The extracted solution was centrifuged at 13,000 rpm for 15 min and the supernatant was used for the measurement of fluorescence intensity (excitation at 485 nm and emission at 538 nm) with the Gemini EM fluorescence microplate reader (Molecular Devices, Sunnyvale, CA, USA).

### 2.11. The 3D-reconstructed Human Skin Models

A 3D-reconstructed human skin model (Neoderm ED^®,^) in a 12-well plate format, produced by culturing human epidermal keratinocytes on top of human dermal fibroblasts in an air-medium interface (air-lift culture) for 12 days, was purchased from TEGO science (Seoul, Korea). The skin model was air-lift cultured for an additional 1 day in this laboratory, at 37 °C in humidified air containing 5% carbon dioxide. The skin model were then treated with 200 μg mL^−1^ PM10 in the presence or absence of 20 μM dieckol for 48 h. The skin model was fixed in 4% paraformaldehyde in PBS and embedded in paraffin. The 6 μm thick sections of paraffin blocks were stained with hematoxylin and eosin and observed with an Eclipse 80i microscope (Nikon Instruments Inc., Melville, NY, USA).

### 2.12. Statistical Analysis

Data are expressed as a mean ± standard deviation (SD) of three or more independent experiments. Experimental results were statistically analysed using SigmaStat v.3.11 software (Systat Software Inc., San Jose, CA, USA), by one-way analysis of variance (ANOVA), followed by Dunnett’s test comparing all treatment groups to a single control group. A *p*-value of less than 0.05 was considered statistically significant. 

## 3. Results

### 3.1. PM10 Induces Cytotoxicity and PGE_2_ Release of Keratinocytes 

**Figure 1:**
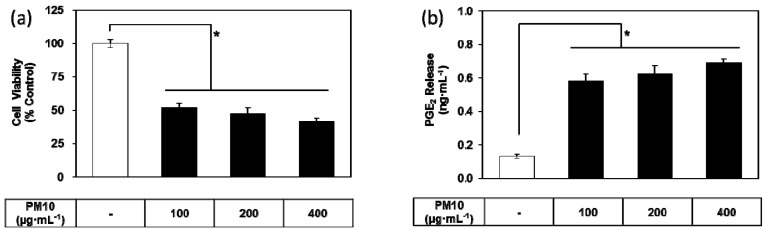
Effects of particulate matter less than 10 microns in diameter (PM10) on the viability and prostaglandin E_2_ (PGE_2_) release of HaCaT keratinocytes. Cells were treated with varying concentrations of PM10 for 48 h for the viability assay (**a**) and the PGE_2_ assay (**b**). Control cells were treated with phosphate-buffered saline. Data are presented as mean ± standard deviation (SD) (*n* = 4). All treatments were compared to the control using one-way analysis of variance (ANOVA) followed by Dunnett’s test. * *p* < 0.05.

To examine whether airborne PM10 causes cytotoxicity and inflammation, HaCaT cells were exposed to PM10 in vitro. PM10 treatments at 100 to 400 μg mL^−1^ for 48 h decreased cell viability (Figure 1a). Conditioned cell culture media were used for the determination of PGE_2_. PGE_2_ production increased in the cells exposed to PM10 at 100 to 400 μg mL^−1^ for 48 h (Figure 1b).

### 3.2. Effects of Marine Alga Extracts on PM10-induced Cytotoxicity

**Figure 2:**
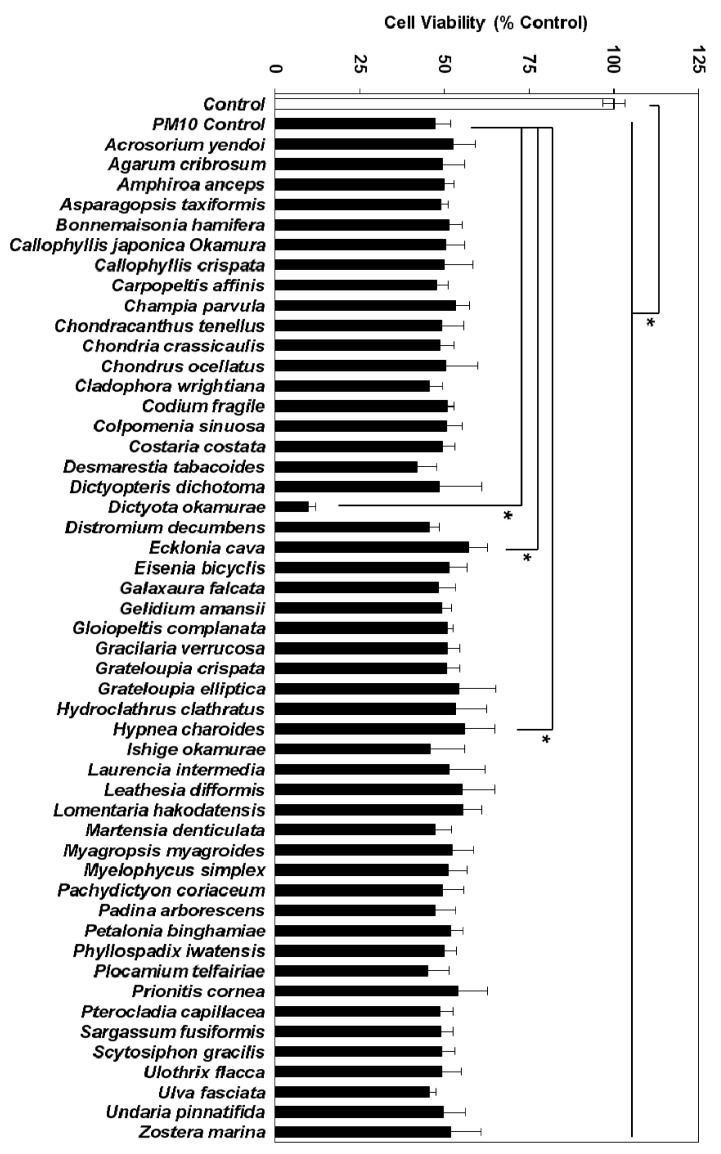
Effects of marine alga extracts on the viability of HaCaT keratinocytes exposed to PM10. Cells were treated with 200 μg mL^−1^ PM10 for 48 h in the presence or absence of each extract at 50 μg mL^−1^. Data are presented as a mean ± SD (*n* = 4). All treatments (50 extracts) were compared to the PM10 control using one-way ANOVA followed by Dunnett’s test. * *p* < 0.05.

To identify the marine alga extracts that alleviated the cytotoxic effects of PM10, HaCaT cells were exposed to PM10 at 200 μg mL^−1^ with or without each alga extract at 50 μg mL^−1^ for 48 h. Of the 50 marine alga extracts, the extract of *E. cava* showed the most protective effects, followed by the extract of *Hypnea charoides* Lamouroux (Figure 2). *E. cava* extract was thus chosen for further study.

### 3.3. Effects of E. cava Extract on PM10-induced Cytotoxicity and PGE_2_ Release

**Figure 3:**
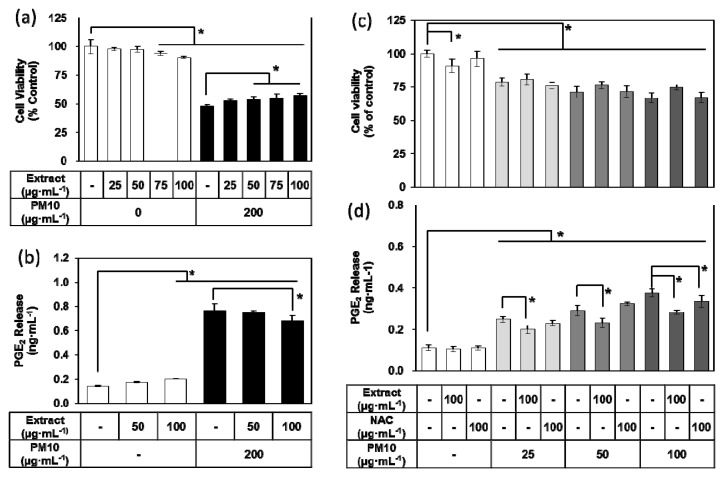
Effects of *Ecklonia cava* extract on the viability and PGE_2_ release of HaCaT keratinocytes exposed to PM10. Cells were treated with PM10 in the absence or presence of *E. cava* extract or N-acetyl cysteine (NAC) for 48 h for the viability assay (**a,c**) and for the PGE_2_ assay (**b,d**). Data are presented as a mean ± SD (*n* = 4). All treatments were compared to the PM10 control using one-way ANOVA followed by Dunnett’s test. * *p* < 0.05.

To examine the effects of *E. cava* extract on cell viability and inflammatory responses in HaCaT keratinocytes exposed to PM10, cells were treated with the extract at concentrations ranging from 25 to 100 μg mL^−1^ with or without 200 μg mL^−1^ PM10. *E*. *cava* extract decreased cell viability and increased PGE_2_ release to a small degree at high concentrations but rescued the cell viability and attenuated the PGE_2_ release stimulated by PM10 in a dose-dependent manner (Figure 3a–b). In additional experiments, cells were treated with 100 μg mL^−1^ of *E. cava* extract and concentrations of PM10 ranging from 25 to 100 μg mL^−1^. NAC was also tested at 100 μg mL^−1^ as a positive control antioxidant. *E. cava* extract-treated cells demonstrated more cell viability than non-treated controls or NAC-treated positive control under various PM10-exposed conditions, although differences among the test, control, and positive control groups were not statistically significant (Figure 3c). *E. cava* extract significantly attenuated the PGE_2_ release stimulated by different concentrations (25–100 μg mL^−1^) of PM10, while NAC showed an inhibitory effect only at 100 μg mL^−1^ PM10 (Figure 3d).

### 3.4. Effects of E. cava Extract on the PM10-induced Gene Expression of the Enzymes Involved in the PGE_2_ Synthesis

**Figure 4:**
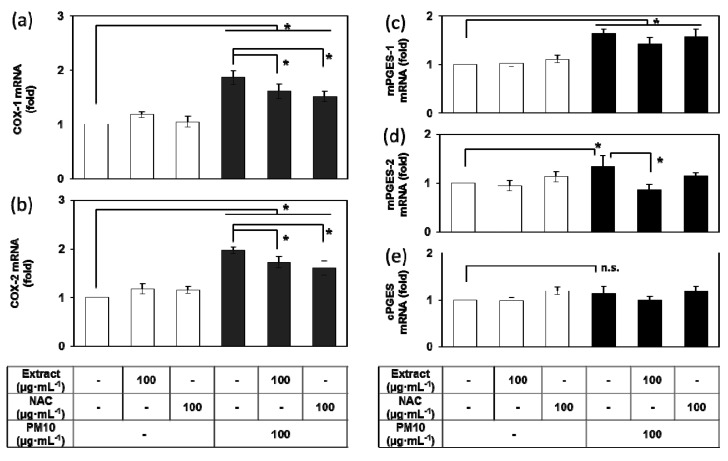
Effects of *Ecklonia cava* extract on the gene expression of enzymes involved in PGE_2_ synthesis in HaCaT keratinocytes exposed to PM10. Cells were treated with PM10 in the presence or absence of *E. cava* extract or NAC for 24 h for the mRNA assays of cyclooxygenase (COX)-1 (**a**), COX-2 (**b**), microsomal prostaglandin E_2_ synthase (mPGES)-1 (**c**), mPGES-2 (**d**), and cytosolic prostaglandin E_2_ synthase (cPGES) (**e**). Data are presented as a mean ± SD (*n* = 3). All treatments were compared to the PM10 control using one-way ANOVA followed by Dunnett’s test. * *p* < 0.05; n.s. was not significant.

Because PM10-induced PGE_2_ release was attenuated by *E. cava* extract, additional experiments were undertaken to determine the mRNA expression levels of COX-1, COX-2, mPGES-1, mPGES-2, and cPGES, the enzymes involved in the PGE_2_ synthesis [42]. PM10 at a concentration of 100 μg mL^−1^ increased the expression of COX-1 and COX-2 at the mRNA level, changes that were significantly attenuated by *E. cava* extract (100 μg mL^−1^) and NAC (100 μg mL^−1^) (Figure 4a–b). PM10 also increased the mRNA levels of mPGES-1 and mPGES-2 but did not increase cPGES mRNA (Figure 4c–e). The PM10-induced increase of mPGES-2 mRNA was attenuated by *E. cava* extract (100 μg mL^−1^). 

### 3.5. Purification of Dieckol from E. cava 

**Figure 5:**
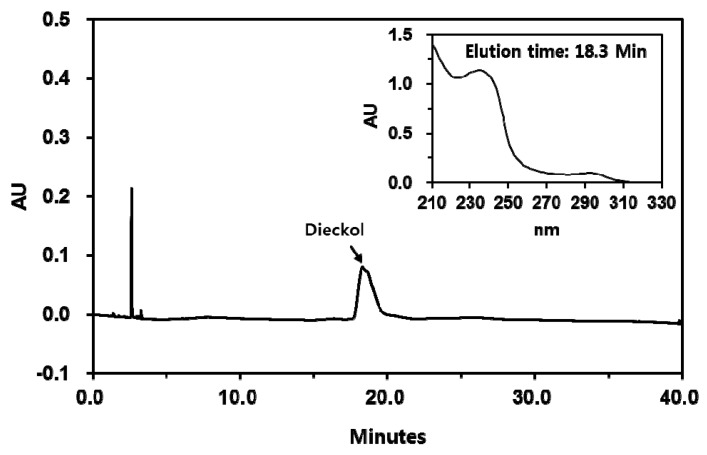
High performance liquid chromatography (HPLC) of dieckol isolated from *Ecklonia cava*. The chromatograms detected at 280 nm are shown. The UV absorption spectrum of a major peak (dieckol) is shown in inset.

Dieckol is a major polyphenolic consituent of *E. cava* that exhibits antioxidant activity [33,43]. Dieckol was purified from *E. cava* extract through solvent fractionation and subsequent chromatography on a normal phase silical gel column and a reversed phase octadecyl silane column. The HPLC profile of purified dieckol is shown in Figure 5.

### 3.6. Analysis of Chemical Structure of Dieckol

**Figure 6:**
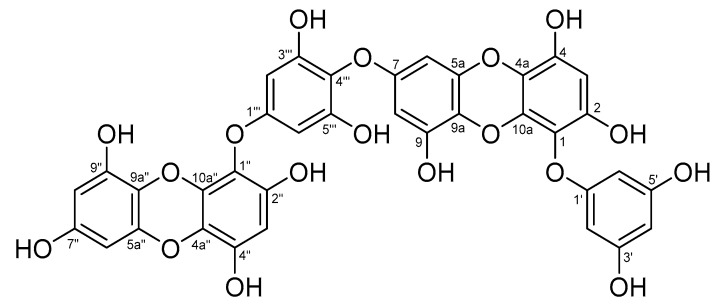
Chemical structure of compound **1** (dieckol).

Compound **1** (Figure 6) was obtained as a pale brown amorphous powder. The molecular formula thereof was determined to be C_36_H_22_O_18_ from ESI-MS, ^1^H-NMR, ^13^C-NMR, and distortionless enhancement by polarization transfer (DEPT) 135 spectral data. The ^1^H-NMR spectrum showed two 1H singlet signals [δ_H_ 6.14 (H-3) and 6.16 (H-3″)], one 2H singlet signal [δ_H_ 6.09 (H-2‴, 6‴)], two sets of meta-coupling doublet signals [δ_H_ 6.06 (d, *J* = 2.8 Hz, H-8)/δ_H_ 6.07 (d, *J* = 2.8 Hz, H-6) and δ_H_ 5.96 (d, *J* = 2.8 Hz, H-6″)/δ_H_ 5.99 (d, *J* = 2.8 Hz, H-8″)], and overlapped AB_2_ system signals [δ_H_ 5.93 (H-2′, 4′, 6′)], respectively. The ^13^C-NMR and DEPT 135 spectra revealed the presence of 11 unsubstituted and 25 oxygen-bearing aromatic carbon signals. The heteronuclear multiple bond correlation (HMBC) spectrum showed long-range couplings, such as H-4′ to C-2′, C-3′, C-5′, and C-6; H-2′(6′) to C-1′, C-3′, and C-4′; H-3 to C-1, C-2, C-4, and C-4a; H-6 to C-5a, C-7, and C-9a; H-8 to C-7, C-9, and C-9a; H-2‴(6‴) to C-1‴, C-3‴, C-4‴, and C-5‴; H-3″ to C-1″, C-2″, C-4″, and C-4a″; H-6″ to C-5a″, C-7″, and C-9a″; and H-8″ to C-7″, C-9″, and C-9a″. Compound **1** was determined to be dieckol on the basis of the above data and comparison with dieckol values in the literature [43,44].

### 3.7. Effects of Dieckol on PM10-induced Cytotoxicity and PGE_2_ Release of Keratibnocytes

**Figure 7:**
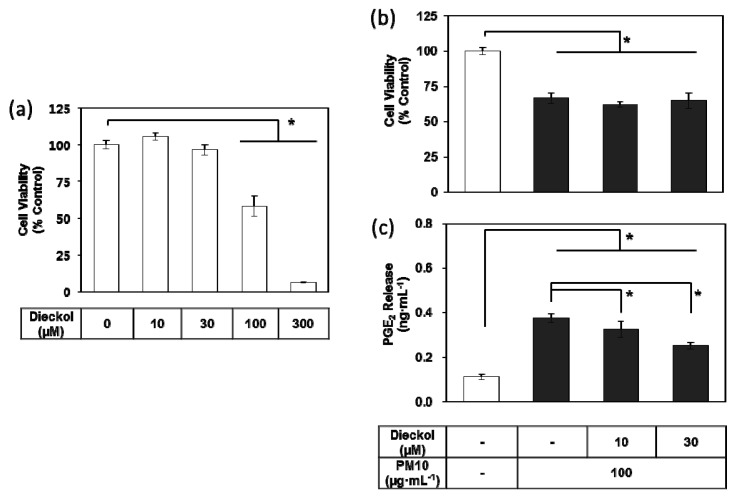
Effects of dieckol on the viability and PGE_2_ release of HaCaT keratinocytes exposed to PM10. Cells were treated with dieckol at varied concentrations for 48 h for the viability assay (**a**). Cells were treated with 100 μg mL^−1^ PM10 in the presence or absence of dieckol at indicated concentrations for 48 h for the viability assay (**b**) and the PGE_2_ assays (**c**). Data are presented as a mean ± SD (*n* = 4). All treatments were compared to the PM10 control using one-way ANOVA followed by Dunnett’s test. * *p* < 0.05.

Dieckol did not change the viability of the HaCaT cells at the tested concentrations up to 30 μM, but it showed toxic effects at concentrations above 100 μM (Figure 7a). In the subsequent experiments, dieckol was used at 10–30 μM, to remain within a non-toxic concentration range. Dieckol attenuated PGE_2_ release in keratinocytes exposed to PM10 in a dose-dependent manner, although it did not rescue cell viability (Figure 7b–c). 

### 3.8. Effects of Dieckol on the PM10-Induced ROS Production and the PM10-Induced Gene Expression of the Enzymes Involved in the PGE_2_ Synthesis.

**Figure 8:**
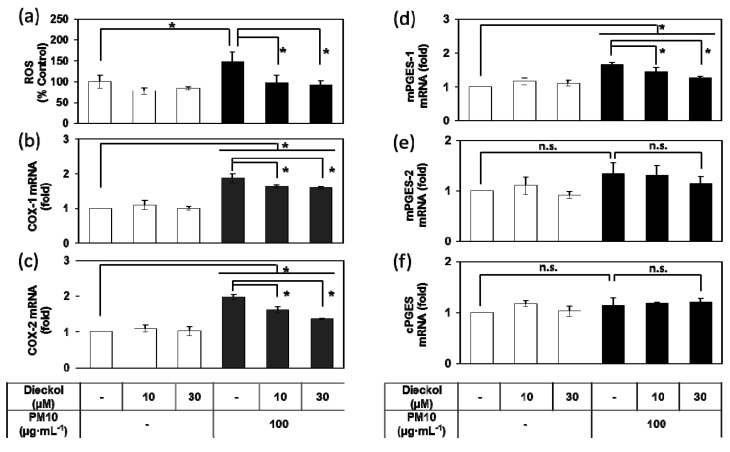
Effects of dieckol on the production of reactive oxygen species (ROS) and the gene expression of enzymes involved in PGE_2_ synthesis in HaCaT keratinocytes exposed to PM10. Cells were treated with 100 μg mL^−1^ PM10 in the presence or absence of dieckol at the indicated concentrations for 30 min for the ROS assay (**a**), and for 24 h for the mRNA assays for COX-1 (**b**), COX-2 (**c**), mPGES-1 (**d**), mPGES-2 (**e**), and cPGES (**f**). Data are presented as a mean ± SD (*n* = 4 for a and *n* = 3 for others). All treatments were compared to PM10 control using one-way ANOVA followed by Dunnett’s test. * *p* < 0.05; n.s. was not significant.

PM10 treatment of HaCaT cells increased ROS production, and the PM-induced change were attenuated by dieckol (Figure 8a). In addition, dieckol attenuated the mRNA expression of COX-1, COX-2, and mPGES-1 induced by PM10 (Figure 8b–f).

### 3.9. Protective Effects of Dieckol against PM10 in a 3D-reconstructed Skin Model

**Figure 9:**
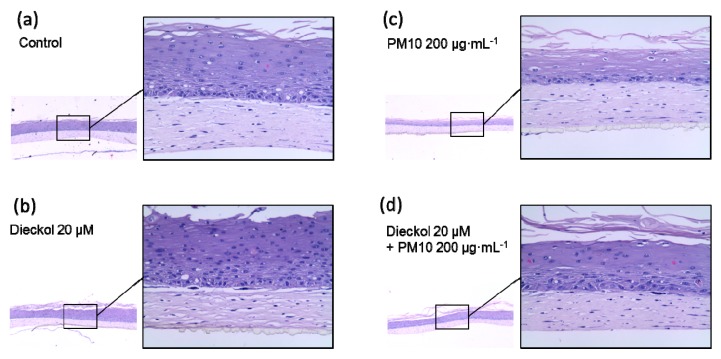
Protective effect of dieckol against PM10 in a skin model. 3D-reconstructed human skin models were treated for 48 h with vehicles (**a**), 200 μg mL^−1^ PM10 (**b**), 20 μM dieckol (**c**), or 20 μM dieckol plus 200 μg mL^−1^ PM10 (**d**). Representative images of the tissue sections stained with hematoxylin and eosin are shown. The magnifications for the small and the enlarged images of each tissue section are × 40 and × 200, respectively.

The protective effects of dieckol against PM10 were further studied using a 3D-reconstructed skin model (Figure 9a–d). The tissue sections stained with hematoxylin and eosin showed morphological differences between the control and PM10-treated cells. PM10 treatment deceased the number of the intact cells at the upper epidermal layer. PM10 tended to decrease the thickness of the epidermal layer, but the change was statistically insignificant. Dieckol itself did not induce significant morphological changes and partially attenuated the morphological changes induced by PM10.

## 4. Discussion

Marine algae have attracted increasing attention as a potential resource for cosmeceutical ingredients [34,45]. *E. cava* is a rich source of phlorotannins, a unique group of polyphenol compounds found in marine brown algae [25,46]. The total phenolic content of *E. cava* extract was estimated to be the highest of the 50 marine plants tested in our previous study [33]. In the present study, of the 50 marine alga extracts tested, *E. cava* extract was the most protective against PM10 toxicity in HaCaT keratinocytes. *E. cava* extract attenuated PGE_2_ production in cells exposed to varying concentrations of PM10 more effectively than NAC, a positive control antioxidant. Dieckol purified from *E. cava* extract also exhibited inhibitory activity against PM10-induced PGE_2_ production. 

The synthesis of PGE_2_ begins with the production of arachidonic acid from membrane phospholipids by the enzymatic action of phospholipase A_2_, followed by the conversion of arachidonic acid to PGG_2_ and then to PGH_2_ by reactions catalyzed by COX-1 and COX-2 [42]. Both isoforms are present in many normal human tissues, and both isoforms are upregulated in a variety of pathological conditions [47]. PGE_2_ synthesis from PGH_2_ is catalyzed by mPGES-1, mPGES-2, and cPGES [48]. Of these isoforms, mPGES-1 is considered responsible for the increased PGE_2_ synthesis during inflammation [49]. 

**Figure 10:**
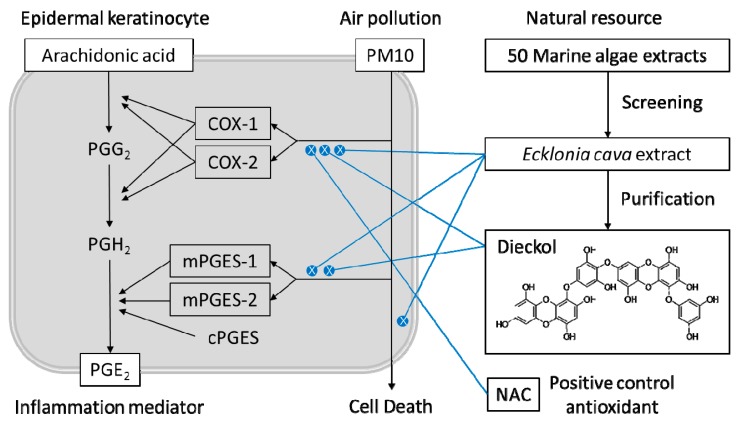
Summary. *Ecklonia cava* extract and its component, dieckol, can alleviate PM10-induced PGE_2_ production in keratinocytes through the inhibition of COX-1, COX-2, mPGES-1, and/or mPGES-2 gene expression.

Previous studies have shown that dieckol and phlorotannins–rich brown alga extracts attenuated PGE_2_ production and COX-2 expression in lipopolysaccharide (LPS)-stimulated RAW 264.7 murine macrophage cells [43], in LPS-stimulated murine BV2 microglia [50], and in UVB radiation-induced skin carcinogenesis in SKH-1 mice [51]. In the present study, PM10 increased the gene expression of both COX-1 and COX-2 in keratinocytes, and these PM-induced COX-1 and COX-2 expressions were ameliorated by *E. cava* extract and dieckol, as well as by NAC (positive control antioxidant). In addition, PM10 increased the expression of mPGES-1 and mPGES-2, and PM10-induced mPGES-2 expression was reduced by *E. cava* extract. Dieckol attenuated the expression of mPGES-1 stimulated by PM10. This suggests that the *E. cava* extract and dieckol can alleviate PM10-induced PGE_2_ production, at least partially, through the inhibition of COX-1, COX-2, mPGES-1, and/or mPGES-2 gene expression (Figure 10). The present study showed that dieckol alleviated the PM-induced inflammatory responses of keratinocytes and PM-induced morphological changes in a 3D-reconstructed skin model. Future studies are warranted to examine clinical efficacy. 

Although the composition of airborne PM differs depending on location, altitude, and season, it nearly always contains toxic components, such as heavy metals and polycyclic hydrocarbons that exert pro-oxidative and pro-inflammatory activity in exposed tissues [52,53,54,55]. PM10 causes the production of ROS through the aryl hydrocarbon receptor/NADPH oxidase-dependent pathway [20,56,57,58]. Our recent study also suggested that dual oxidase 2 plays a critical role in ROS production in keratinocytes exposed to PM [59]. Thus, antioxidants have the potential to alleviate adverse skin reactions that arise from PM exposure [24]. 

In the previous study, pomegranate peel extract and punicalagin attenuated PM10-induced inflammatory monocytes adhesion to endothelial cells [55]. Epigallocatechin gallate derived from green tea and punicalagin reduced the PM10-induced expression of inflammatory cytokines, such as the tumor necrosis factor (TNF)-α, interleukin (IL)-1β, IL-6, and IL-8 [54]. Resveratrol and resveratryl triacetate inhibited the expression of PM10-induced IL-6 in keratinocytes [60]. In addition, *E. cava* extract attenuated cellular lipid peroxidation in keratinocytes induced by PM10 [33]. Dieckol, one of the major phlorotannins of *E. cava,* attenuated cellular lipid peroxidation and the expression of inflammatory cytokines TNF-α, IL-1β, IL-6, and IL-8 at the mRNA and protein levels in human epidermal keratinocytes exposed to PM10 [33]. Taken together, data from these previous studies and the current study suggest that polyphenol-rich plant extracts and individual polyphenolic compounds can mitigate oxidative stress and inflammation in the skin that occur as a result of exposure to airborne PM10. 

It was previously shown that PM-induced cellular ROS production was attenuated by various antioxidants, such as NAC, apocynin, resveratrol, resveratryl triacetate, punicalagin, (−)-epigallocatechin gallate, and eupafolin [22,54,55,60]. In the present study, dieckol was shown to attenuate the PM-induced ROS production in keratinocytes. PM-derived ROS can lead to the activation of the mitogen activated protein kinase (MAPK) family including extracellular signal regulated kinase (ERK), c-Jun N-terminal kinase (JNK), and p38 kinase, and the stimulation of nuclear factor-kappa B (NF-κB) signaling pathway, leading to the activation of redox-sensitive transcription factors activator protein 1 (AP-1) and NF-κB [61,62]. The expression of COX-2 mRNA is regulated by several transcription factors including the cyclic-AMP response element binding protein, NF-κB and the CCAAT-enhancer binding protein, which are activated by various MAPKs and other protein kinases [63]. PM stimulates MAPKs such as ERK, p38 and JNK in keratinocytes which ultimately induce the expression of COX-2 [20,64]. Therefore, a variety of redox-sensitive signaling pathways are involved in the regulation of PGE_2_ production in response to PM, and antioxidants contained in *E. cava,* such as dieckol, are assumed to interfere with these multiple signaling pathways, attenuating PM-induced PGE_2_ production. Further studies are needed to verify this notion and to examine in vivo efficacy of dieckol.

## 5. Conclusions

In conclusion, this study demonstrated that that airborne PM10 stimulated COX-1, COX-2, mPGES-1, and mPGES-2 gene expression, and thereby PGE_2_ production, in keratinocytes. *E. cava* extract and dieckol were shown to alleviate PM10-induced PGE_2_ production through the inhibition of gene expression of COX-1 COX-2, mPGES-1, and/or mPGES-2. *E. cava* extract and dieckol are potentially useful natural cosmetic ingredients for counteracting the pro-inflammatory effects of airborne PM on the skin. 

## Figures and Tables

**Table 1:** ^1^H- and ^13^C-nuclear magnetic resonance (NMR) spectroscopic data for compound **1** (dieckol) *^a^*.

Position	δ_H_ Multiplicity, (*J* Hz)	δ_C_ Multiplicity	Position	δ_H_ Multiplicity, (*J* Hz)	δ_C_ Multiplicity
1		124.7 ^c^ s	1″		124.8 *^c^* s
2		147.5 s	2″		147.4 s
3	6.14 s	99.5 d	3″	6.16 s	99.6 d
4		143.4 s	4″		143.5 s
4a		125.8 *^d^* s	4a″		125.7 *^d^* s
5a		143.3 s	5a″		144.4 s
6	6.07 d (2.8)	96.0 d	6″	5.96 d (2.8)	95.9 d
7		156.1 s	7″		154.7 s
8	6.06 d (2.8)	99.9 d	8″	5.99 d (2.8)	100.0 d
9		147.1 s	9″		147.3 s
9a		126.3 s	9a″		125.0 s
10a		138.6 s	10a″		138.8 s
1′		162.0 s	1‴		157.9 s
2′, 6′	5.93 *^b^*	95.5 d	2‴, 6‴	6.09 s	96.3 d
3′, 5′		160.3 s	3‴, 5‴		152.5 s
4′	5.93 *^b^*	97.8 d	4‴		126.6 s

*^a^* Measured at 700 and 175 MHz; obtained in CD_3_OD with tetramethylsilane (TMS) as an internal standard. Assignments were based on ^1^H–^1^H correlation spectroscopy (COSY), heteronuclear single quantum correlation (HSQC), and heteronuclear multiple bond correlation (HMBC) experiments. *^b^* Overlapped with other signals. *^c–d^* Interchangeable signals

**Table 2:** Sequences of primers used for the quantitative reverse-transcriptase polymerase chain reaction (qRT-PCR) of gene transcripts.

Gene Name	GenBank Accession Number	Primer Sequences	Ref.
Cyclooxygenase 1 (COX-1); Prostaglandin-endoperoxide synthase 1 (PTGS1)	NM_000962.4	Forward: 5′-CAGAGCCAGATGGCTGTGGG-3′ Reverse: 5′-AAGCTGCTCATCGCCCCAGG-3′	[38]
Cycloxygenase 2 (COX-2); Prostaglandin-endoperoxide synthase 2 (PTGS2)	NM_000963.3	Forward: 5′-CTGCGCCTTTTCAAGGATGG-3′ Reverse: 5′-CCCCACAGCAAACCGTAGAT-3′	[39]
Microsomal prostaglandin E synthase 1 (mPGES-1); Prostaglandin E synthase 1 (PTGES 1)	NM_004878.5	Forward: 5′-AACCCTTTTGTCGCCTG-3′ Reverse: 5′-GTAGGCCACGGTGTGT-3′	[40]
Microsomal prostaglandin E synthase 1 (mPGES-2); Prostaglandin E synthase 2 (PTGES 2)	NM_025072.7	Forward: 5′-GAAAGCTCGCAACAACTAAAT-3′ Reverse: 5′-CTTCATGGCTGGGTAGTAG-3′	[40]
Cytosolic prostaglandin E synthase (cPGES); Prostaglandin E synthase 3 (PTGES3),	NM_006601.6	Forward:5′-ATAAAAGAACGGACAGATCAA-3′ Reverse:5′-CACTAAGCCAATTAAGCTTTG-3′	[40]
GAPDH (glyceraldehyde 3-phosphate dehydrogenase)	NM_002046.3	Forward: 5′-ATGGGGAAGGTGAAGGTCG-3′ Reverse: 5′-GGGGTCATTGATGGCAACAA-3′	[33]
